# The risk of twin pregnancies should be minimized in patients with a unicornuate uterus undergoing IVF-ET

**DOI:** 10.1038/s41598-020-62311-5

**Published:** 2020-03-27

**Authors:** Yan Ouyang, Pei Cai, Fei Gong, Ge Lin, Jiabi Qin, Xihong Li

**Affiliations:** 1 College of Life Science, Hunan Normal University, Changsha, China; 2 Reproductive and Genetic Hospital of CITIC-Xiangya, Changsha, China; 3 Clinical Research Center For Reproduction and Genetics in Hunan Province, Changsha, China; 4 Institute of Reproductive and Stem Cell Engineering, Central South University, Changsha, China; 5 Department of Epidemiology and Health Statistics, Xiangya School of Public Health, Central South University, Changsha, China

**Keywords:** Ultrasonography, Quality of life, Outcomes research

## Abstract

Unicornuate uteri are associated with infertility, miscarriage, preterm delivery and even uterine rupture. The aim of this research was to investigate the effects of unicornuate uterine anomaly on twin pregnancies after *in vitro* fertilization-embryo transfer (IVF-ET). A total of 206 women with unicornuate uteri (A singleton, B selective reduction (SR) of twins to a singleton, C twins) and 314 women with normal uteri (D SR of twins to a singleton, E twins) who delivered at ≥22 weeks were included. C was associated with a significantly lower live birth rate (adjusted odds ratio (aOR) 0.08, 95% confidence interval (CI), 0.01–0.69) and higher risks of preterm delivery (aOR 11.63, 95% CI, 4.85–27.92), perinatal mortality (aOR 11.43, 95% CI, 1.44–90.57) and low birth weight (aOR 5.92, 95% CI 1.94–18.06) than A, a 15-fold greater risk of preterm delivery (aOR 15.54, 95% CI 3.09–78.28) than B and a greater risk of preterm delivery (aOR 2.76, 95% CI 1.33–5.73) than E. After SR to a singleton, the perinatal outcomes were statistically similar between B and D. These results showed that the risk of twin pregnancies should be minimized in patients with unicornuate uterine anomaly undergoing IVF-ET.

## Introduction

Congenital uterine anomalies are estimated to occur in 8–13% of infertile women^1,2^. Unicornuate uteri, which result from the partial or complete failure of one paramesonephric duct to develop, account for 5–13%^3^ of all congenital uterine anomalies. According to previous studies^4,5^ unicornuate uteri are associated with adverse reproductive outcomes, including infertility, miscarriage, preterm delivery, foetal intrauterine growth retardation and even uterine rupture.

With the rapid development of assisted reproductive technology (ART), the rates of multiple pregnancies have increased significantly^6,7^. Twin pregnancies account for the vast majority and are reported to carry 4–5 times more risk than singleton pregnancies. Data have shown that selective reduction (SR) of twins to a singleton may yield better outcomes^8,9^. Therefore, patients who conceive twin pregnancies via *in vitro* fertilization-embryo transfer (IVF-ET) are often faced with the difficult choice of whether to undergo SR^10^.

However, little is known about the effects of unicornuate uterine anomaly on twin pregnancies and the outcomes of SR to a singleton after IVF-ET. Thus, in this study, we compared the perinatal outcomes of twin pregnancies and SR to a singleton between women with and without a unicornuate uterus. The perinatal outcomes of singleton pregnancies, SR to a singleton and twin pregnancies were also compared among women with a unicornuate uterus to investigate the effects of unicornuate uterine anomaly on twin pregnancies after IVF-ET.

## Materials and Methods

### Patient selection

This retrospective, single-centre cohort study was approved by the ethics committee of the Reproductive and Genetic Hospital of CITIC-Xiangya. Informed consent was not required because the data were collected retrospectively in an anonymous manner.

Recruitment was conducted from January 2012 to December 2014. Two hundred and six women who were diagnosed with a unicornuate uterus and 82 women who had a normal uterus and received SR of twins to a singleton were enrolled. Because the number of twin pregnancies (without reduction) in patients with a normal uterus was very high during this period, we selected twin pregnancies (without reduction) during the middle 3 months (January 2013 to March 2013, n = 232).

**Figure 1:**
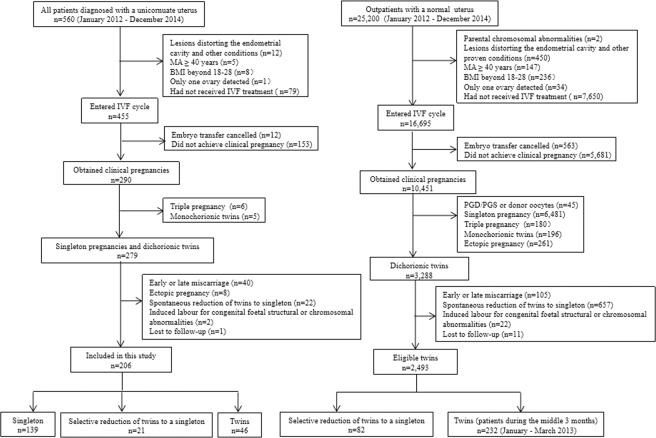
Flow chart of patient inclusion. Abbreviations: MA, maternal age; BMI, body mass index; IVF, *in vitro* fertilization; PGD, preimplantation genetic diagnosis; PGS, preimplantation genetic screening.

All enrolled patients successfully achieved clinical pregnancies and delivered at ≥22 gestational weeks after IVF-ET. To avoid selection bias, only the first pregnancy of each patient was considered. Patients were excluded if any of the following criteria were met: maternal age (MA) ≥ 40 years old; body mass index (BMI) outside the range of 18–28; only one ovary detected; uterine fibroids or polyps distorting the endometrial cavity; received donor oocytes; preimplantation genetic diagnosis (PGD)/preimplantation genetic screening (PGS); parental chromosomal abnormalities; spontaneous reduction; monochorionic twin or triplet pregnancies; early or late miscarriage; ectopic pregnancy; or induced labour. The flow of patient inclusion is shown in Fig. 1.

According to the gestational number, women with a unicornuate uterus were divided into singleton (A, n = 139), SR of twins to a singleton (B, n = 21) and twin (C, n = 46) pregnancy groups; women with a normal uterus were divided into SR of twins to a singleton (D, n = 82) and twin (E, n = 232) pregnancy groups. The perinatal outcomes were compared among these groups.

### Ultrasound diagnosis and IVF procedure

Three-dimensional transvaginal ultrasonography (3D-TVS, GE VOLUSON E8/730, General Electric Tech Co., Ltd., New York, USA) was performed as a routine step to analyse the uterine anatomy before beginning the IVF cycle. The diagnosis of a unicornuate uterus was based on the European Society of Human Reproduction and Embryology and the European Society for Gynaecological Endoscopy (ESHRE/ESGE) classification system^3^. Hysterosalpingography, hysteroscopy and/or laparoscopy, or laparotomy were further used to confirm the diagnosis of a uterine anomaly. Immunological, genetic, endocrine and blood test results were also routinely recorded. All enrolled women had normal chromosomal karyotypes, and no other urinary tract malformations were detected.

During the IVF-ET procedure, fresh or frozen embryos were transferred, and fertilization was conducted using either standard IVF or intracytoplasmic sperm injection. One to three good-quality embryos (grade 1 or 2 embryos with at least seven blastomeres) were transferred at the day-3 stage. The embryo scoring method was described in our previous study^11^.

Serum human chorionic gonadotropin (hCG) levels were measured on day 14, and TVS was performed 4 weeks after ET. Clinical pregnancy was diagnosed if a gestational sac was observed, and a viable pregnancy was confirmed when cardiac activity was detected. If more than one gestational sac was found in the uterus, a twin or multiple pregnancy was diagnosed.

### The SR strategy

In our centre, patients with multiple pregnancies were comprehensively counselled about the high risks and offered the option of foetal reduction. Women with unicornuate uteri with twin pregnancies were informed about the possible adverse effects of the unicornuate uterus on pregnancy outcome, and SR to a singleton pregnancy was recommended. If the couple insisted on maintaining two foetuses, informed consent was obtained.

All SR procedures were conducted transabdominally at 11–13^+6^ gestational weeks and were carried out by an experienced operator in our centre. Before the reduction procedure, informed consent was obtained from all patients, and an ultrasound scan was performed to confirm the number, locations, sizes and cardiac activity of the foetuses. In general, foetuses with growth retardation or structural abnormalities were chosen for reduction. For twin pregnancies with normal growth, the foetus farther away from the cervix was typically selected for reduction, which was accomplished by injecting potassium chloride intracardially. The arrest of cardiac activity was confirmed at the end of the procedure. Follow-up TVS examinations were arranged on the 2^nd^ day and the 4^th^ day to confirm the success of the reduction procedure.

### Follow-up and main outcome measures

All patients were tracked until the end of pregnancy by a specified team at our centre. The delivery mode, gestational age (GA) at delivery, survival of the foetus(es) and neonatal birth weight were collected via a telephone call or by fax. The main outcome measures were defined as follows. A preterm delivery was defined as a birth occurring after 22 weeks and before 37 completed weeks of GA. A live birth was defined as the complete expulsion or extraction from a woman of a product of fertilization after 22 completed weeks of GA that, after this separation, breathed or showed any other evidence of life, irrespective of whether the umbilical cord had been cut or the placenta was attached. Perinatal mortality was foetal or neonatal death occurring during late pregnancy (at 22 completed weeks of GA and later), during childbirth, or up to 7 completed days after birth^12^. Low birth weight (LBW) was defined as a birth weight <2500 g and very low birth weight (VLBW) as a birth weight <1500 g^12,13^. The GA was calculated by subtracting the date of embryo transfer from the date of birth and adding 17 days.

### Statistical analysis

Measurements are expressed as the means ± standard deviation, and the enumerated data are expressed as the rates (percentages). Student’s t-test was used to analyse differences between means. The chi-square test or Fisher’s exact test was used to determine significant differences between percentages. Odds ratios (ORs) and their 95% confidence intervals (CIs) were used to demonstrate the level of association. The unadjusted ORs and adjusted ORs (aORs) were calculated by logistic regression. All characteristics that were significantly different between groups in the univariate analysis were included in the multivariable logistic regression. A p value <0.05 was considered significant. All analyses were performed using SPSS software version 17.0 (SPSS, Inc., Chicago, IL, USA).

## Results

From January 2012 to December 2014, 560 patients were diagnosed with unicornuate uteri. Among them, 455 eligible patients entered IVF cycles. Embryo transfer was cancelled in 12 cases, and clinical pregnancy was achieved in 290 cases (65.5%, 290/443). After patient exclusion, the data from 206 patients with unicornuate uteri were ultimately analysed. During the same time period, 82 cases of SR of twins to a singleton and 232 cases of twin pregnancies in women with a normal uterus were also selected (Fig. 1).

### Patient characteristics

#### Comparisons among the unicornuate uterine groups

The singleton (A) and twin (C) groups were significantly different in terms of the number of transferred embryos (p = 0.003), the 14-day hCG level (p < 0.001) and the insemination method (p = 0.007).

When the twin group (C) was compared to the SR group (B), all characteristics were similar (p > 0.05), with the exception of follicle-stimulating hormone (FSH, p = 0.014) levels and infertility type (p = 0.008). When the SR group (B) was compared to the singleton group (A), the infertility type (p = 0.036) and 14-day hCG (p < 0.001) were significantly different, but the other characteristics were similar (p > 0.05) (Table 1).

**Table 1:** Comparisons of patient characteristics.

Characteristics	Unicornuate uterus			Normal uterus		Univariate analysis				
	Singleton (n = 139) A	SR of twins to a singleton (n = 21) B	Twins (n = 46) C	SR of twins to a singleton (n = 82) D	Twins (n = 232) E	*P* (A vs. B)	*P* (A vs. C)	*P* (B vs. C)	*P* (D vs. B)	*P* (E vs. C)
**Maternal age (years)**	29.5 ± 4.4	28.1 ± 2.9	29.2 ± 3.7	31.9 ± 3.7	29.7 ± 3.7					
≤24	11(7.9)	2(9.5)	5(10.9)	4(4.9)	15(6.5)	0.255	0.209	0.614	**0.002**	0.680
25–29	69(49.6)	12(57.1)	19(41.3)	17(20.7)	97(41.8)					
30–34	39(28.1)	7(33.3)	19(41.3)	40(48.8)	91(41.8)					
≥35	20(14.4)	0	3(6.5)	21(25.6)	23(9.9)					
**BMI (kg/m**^2^**)**	21.6 ± 3.8	21.4 ± 2.5	21.2 ± 2.9	21.2 ± 2.8	21.2 ± 2.6					
<18.5	24(17.5)	3(14.3)	6(13.0)	12(14.6)	34(14.7)	1.000	0.276	0.693	0.882	0.874
18.5–23.9	87(63.5)	14(66.7)	35(76.1)	58(70.7)	168(72.4)					
≥24	26(19.0)	4(19.0)	5(10.9)	12(14.6)	30(12.9)					
**FSH (mIU/mL)**	6.1 ± 3.5	5.5 ± 3.2	6.1 ± 1.3	4.9 ± 3.1	5.8 ± 1.7					
≤1.36	8(5.8)	3(14.3)	0	20(24.4)	0	0.209	0.283	**0.014**	0.606	0.517
1.37–9.9	127(91.4)	17(81.0)	45(97.8)	59(72.0)	229(98.7)					
≥10.0	4(2.9)	1(4.8)	1(2.2)	3(3.7)	3(1.3)					
**Infertility duration (years)**	4.8 ± 3.3	3.6 ± 2.1	5.0 ± 3.3	4.79 ± 3.12	5.7 ± 3.3					
≤3	60(43.2)	11(52.4)	18(39.1)	35(42.7)	66(28.4)	0.235	0.819	0.175	0.246	0.271
4–6	43(30.9)	8(38.1)	14(30.4)	25(30.5)	96(41.4)					
≥7	36(25.9)	2(9.5)	14(30.4)	22(26.8)	70(30.2)					
**Previous miscarriage**										
No	119(85.6)	19(90.5)	35(76.1)	73(89.0)	207(89.2)	0.741	0.134	0.203	0.848	**0.015**
Yes	20(14.4)	2(9.5)	11(23.9)	9(11.0)	25(10.8)					
**Transfer cycle**	1.1 ± 0.4	1.1 ± 0.3	1.2 ± 0.5	1.23 ± 0.61	1.2 ± 0.4					
1	121(87.1)	19(90.5)	41(89.1)	68(84.0)	200(86.2)	1.000	0.711	1.000	0.452	0.594
≥2	18(12.9)	2(9.5)	5(10.9)	13(16.0)	32(13.8)					
**Number of retrieved oocytes**	8.8 ± 6.3	10.6 ± 5.5	11.3 ± 5.9	10.2 ± 6.6	9.7 ± 6.8					
≤10	66(47.5)	9(42.9)	16(34.8)	30(36.6)	103(44.4)	0.692	0.133	0.526	0.597	0.229
>10	73(52.5)	12(57.1)	30(65.2)	52(63.4)	129(55.6)					
**EM thickness on transfer day (mm)**	12.5 ± 2.2	12.9 ± 2.2	12.2 ± 2.1	12.5 ± 2.2	12.7 ± 2.2					
≤7.9	1(0.7)	0	0	0	1(0.4)	1.000	0.851	0.698	0.860	0.609
8–14.9	120(86.3)	18(85.7)	41(89.1)	69(84.1)	194(83.6)					
≥15	18(12.9)	3(14.3)	5(10.9)	13(15.9)	37(16.0)					
**Number of transferred embryos**										
**1**	23(16.5)	0	0	0	0	0.066	**0.003**	1.000	1.000	1.00
**2**	110(79.1)	21(100.0)	44(95.7)	79(96.3)	221(95.3)					
**3**	6(4.3)	0	2(4.3)	3(3.7)	11(4.7)					
**Infertility type**										
Primary	72(51.8)	16(76.2)	19(41.3)	33(40.2)	126(54.3)	**0.036**	0.217	**0.008**	**0.003**	0.107
Secondary	67(48.2)	5(23.8)	27(58.7)	49(59.8)	106(45.7)					
**Cause of infertility**										
Male factor	7(5.0)	0	1(2.2)	3(3.7)	13(5.6)	0.613	0.856	0.810	0.526	0.400
Female factor	83(59.7)	11(52.4)	27(58.7)	48(58.5)	112(48.3)					
Combined female and male factors	47(33.8)	10(47.6)	17(37.0)	31(37.8)	105(45.3)					
Unexplained	2(1.4)	0	1(2.2)	0	2(0.9)					
**14-day hCG (mIU/ml)**	516.1 ± 261.6	1069.6 ± 264.4	1067.0 ± 404.3	967.7 ± 338.6	1015.5 ± 438.6					
<420	59(42.4)	0	1(2.2)	2 (2.4)	14(6.0)	**<0.001**	**<0.001**	1.000	0.202	0.462
≥420	80(57.6)	21(100.0)	45(97.8)	80(97.6)	218(94.0)					
**Insemination method**										
IVF	72(51.8)	16(76.2)	36(78.3)	47(57.3)	190(81.9)	0.136	**0.007**	1.000	0.280	**<0.001**
ICSI	26(18.7)	2(9.5)	4(8.7)	16(19.5)	42(18.1)					
IVF/ICSI	41(29.5)	3(14.3)	6(13.0)	19(23.2)	0(0.0)					

Abbreviations: SR, selective reduction; BMI, body mass index; FSH, follicle-stimulating hormone; EM, endometrium; hCG, human chorionic gonadotropin; IVF, *in vitro* fertilization; ICSI, intracytoplasmic sperm injection.

a. Values are given as numbers (percentages) or mean ± SD, unless otherwise indicated.

#### Comparisons between the unicornuate and normal uterine groups

With the exception of MA (p = 0.002) and infertility type (p = 0.003), all other characteristics were similar (p > 0.05) between the SR groups for women with a unicornuate uterus (B) and women with a normal uterus (D).

For twin pregnancies in women with a unicornuate uterus (C) and women with a normal uterus (E), except for the transfer cycle (p = 0.015) and insemination method (p < 0.001), other factors were all statistically similar (p > 0.05).

### Perinatal outcomes

#### Comparisons among the unicornuate uterine groups

Compared to the singleton group (A), the twin group (C) showed a significantly lower live birth rate (76.1% vs. 99.3%, OR 0.02, 95% CI, 0.00–0.19). In addition, the rates of preterm delivery (67.4% vs. 18.0%, OR 9.42, 95% CI 4.44–20.02), perinatal mortality (21.7% vs. 0.7%, OR, 38.33, 95% CI 5.04–291.43), LBW (63.0% vs. 12.2%, OR, 12.24, 95% CI 6.32–23.71) and VLBW (23.9% vs. 1.4%, OR, 21.53, 95% CI 4.92–94.19) were all significantly higher in the twin group (C) than in the singleton group (A).

The twin group (C) was associated with a markedly lower live birth rate (76.1% vs. 100%, p = 0.048) than the SR group (B). Additionally, significantly higher rates of preterm delivery (67.4% vs. 9.5%, OR, 19.63, 95% CI, 4.04–95.52), perinatal mortality (21.7% vs. 0, p = 0.022), LBW (63.0% vs. 9.5%, OR, 16.21, 95% CI, 3.55–73.90, p < 0.001) and VLBW (23.9% vs. 0, p = 0.012) were observed in the twin group (C).

The caesarean section rate was significantly lower in the SR group (B) than in the singleton group (A) (66.7% vs. 86.3%, OR 0.32, 95% CI 0.11–0.89), but other perinatal outcomes were similar between these two groups (Table 2).

**Table 2:** Comparisons of perinatal outcomes^a^.

Perinatal outcomes	Unicornuate uterus			Normal uterus		A vs. B	A vs. C	B vs. C	D vs. B	E vs. C
	Singleton (n = 139) A	SR (n = 21) B	Twin (n = 46) C	SR (n = 82) D	Twin (n = 232) E	Unadjusted OR (95% CI)	Unadjusted OR (95% CI)	Unadjusted OR (95% CI)	Unadjusted OR (95% CI)	Unadjusted OR (95% CI)
Babies born	139	21	92	82	232					
Live births	138	21	72	81	232					
Live birth rate	138(99.3)	21(100.0)	35(76.1)	81(98.8)	232(100.0)	—	**0.02 (0.00–0.19)**	—	—	—
Preterm delivery rate	25(18.0)	2 (9.5)	31(67.4)	5(6.1)	92(39.7)	0.48 (0.11–2.20)	**9.42 (4.44–20.02)**	**19.63 (4.04–95.52)**	1.62 (0.29–9.01)	**3.15 (1.61–6.15)**
Perinatal mortality	1(0.7)	0	20(21.7)	1(1.2)	0	—	**38.33 (5.04–291.43)**	—	—	—
Caesarean section rate	120(86.3)	14(66.7)	34(73.9)	57(69.5)	211(90.9)	**0.32 (0.11–0.89)**	0.45 (0.20–1.02)	1.42 (0.46–4.35)	0.88 (0.32–2.44)	**0.28 (0.13–0.63)**
Gestational week at delivery (weeks)										
≥37	114(82.0)	19(90.5)	15(32.6)	77(93.9)	139(59.9)	2.08 (0.46–9.53)	**0.11 (0.05–0.23)**	**0.05 (0.01–0.25)**	0.62 (0.11–3.42)	**0.32 (0.17–0.63)**
<37	25(18.0)	2(9.5)	31(67.4)	5(6.1)	93(40.1)	0.48 (0.11–2.20)	**9.42 (4.44–20.02)**	**19.63 (4.04–95.52)**	1.62 (0.29–9.00)	**3.09 (1.58–6.04)**
Live birth weight (g)										
≥2500	122(87.8)	19(90.5)	34(37.0)	78(95.1)	276(59.5)	1.32 (0.28–6.19)	**0.08 (0.04–0.16)**	**0.06 (0.01–0.28)**	0.62 (0.11–3.43)	**0.40 (0.25–0.63)**
LBW < 2500	17(12.2)	2(9.5)	58(63.0)	4(4.9)	188(40.5)	0.76 (0.16–3.53)	**12.24 (6.32–23.71)**	**16.21 (3.55–73.90)**	2.05 (0.35–12.5)	**2.50 (1.58–3.98)**
VLBW < 1500	2(1.4)	0	22(23.9)	0	16(3.4)	—	**21.53 (4.92–94.19)**	—	—	**8.80 (4.41–17.57)**

Abbreviations: SR, selective reduction; OR, odds ratio; CI, confidence interval; LBW, low birth weight; VLBW, very low birth weight.

a. Values are given as numbers (percentages) or mean ± SD, unless otherwise indicated.

#### Comparisons between the unicornuate and normal uterine groups

In women with a twin pregnancy, the presence of a unicornuate uterus (E) was associated with significantly decreased rates of live birth (76.1% vs. 100.0%, P < 0.001) and caesarean section (73.9% vs. 90.9%, OR, 0.28, 95% CI, 0.13–0.63) and markedly increased risks of preterm delivery (67.4% vs. 39.7%, OR, 3.15, 95% CI, 1.61–6.15), perinatal mortality (21.7% vs. 0, P < 0.001), LBW (63.0% vs. 40.5%, OR, 2.50, 95% CI, 1.58–3.98) and VLBW (23.9% vs. 3.4%, OR, 8.80, 95% CI, 4.41–17.57) compared with those in women with a unicornuate uterus (C).

After SR of twins to a singleton, all perinatal outcomes were significantly similar between women with a unicornuate uterus (B) and women with a normal uterus (D) (Table 2).

### Findings from the multiple logistic regression analysis

#### Comparisons among the unicornuate uterine groups

After adjustment, the twin group (C) was associated with a much lower rate of live birth (aOR 0.08, 95% CI, 0.01–0.69; p = 0.021) and significantly higher risks of preterm delivery (aOR 11.63, 95% CI, 4.85–27.92; p < 0.001), perinatal mortality (aOR 11.43, 95% CI, 1.44–90.57; p = 0.021) and LBW (aOR 5.92, 95% CI 1.94–18.06; p = 0.002) than the singleton group (A).

The twin group (C) was associated with a 15-fold greater risk of preterm delivery (aOR 15.54, 95% CI 3.09–78.28; p = 0.001) than the SR group (B). Increased risks were also found for caesarean sections (aOR 6.35, 95% CI 0.69–58.23, p = 0.102) and LBW (aOR 7.27, 95% CI 0.42–124.65; p = 0.171), but the difference was not significant.

The risks of preterm delivery (aOR 0.46, 95% CI 0.10–2.25; p = 0.338) and LBW (aOR 1.19, 95% CI 0.14–9.87; p = 0.871) were statistically similar between the SR group (B) and the singleton group (A); however, the risk of caesarean section was significantly lower in the SR group (B) (aOR 0.18, 95% CI 0.05–0.63; p = 0.007) (Table 3).

**Table 3:** Multiple logistic regression analysis of the risks for perinatal outcomes.

Perinatal outcomes	A vs. B		A vs. C		B vs. C		D vs. B		E vs. C	
	*P*	Adjusted OR (95% CI)^a^	*P*	Adjusted OR (95% CI)^b^	*P*	Adjusted OR (95% CI)^c^	*P*	Adjusted OR (95% CI)^d^	*P*	Adjusted OR (95% CI)^e^
Live birth	—	—	**0.021**	**0.08(0.01–0.69)**	—	—	—	—	—	—
Preterm delivery	0.338	0.46(0.10–2.25)	**<0.001**	**11.63(4.85–27.92)**	**0.001**	**15.54(3.09–78.28)**	0.360	2.61(0.34–20.32)	**0.006**	**2.76(1.33–5.73)**
Perinatal mortality^*^	—	—	**0.021**	**11.43(1.44–90.57)**	—	—	—	—	—	—
Caesarean section^*^	**0.007**	**0.18(0.05–0.63)**	0.753	0.83(0.26–2.60)	0.102	6.35(0.69–58.23)	0.682	1.26(0.41–3.85)	0.112	0.46(0.17–1.20)
LBW < 2500 g^*^	0.871	1.19(0.14–9.87)	**0.002**	**5.92(1.94–18.06)**	0.171	7.27(0.42–124.65)	0.681	1.74(0.13–24.26)	0.158	1.75(0.81–3.80)

Abbreviations: OR, odds ratio; CI, confidence interval; LBW, low birth weight.

a. Adjusted for infertility type and 14-day hCG.

b. Adjusted for no. of transferred embryos, 14-day hCG and insemination method.

c. Adjusted for FSH and infertility type.

d Adjusted for maternal age and infertility type.

e Adjusted for previous miscarriage and insemination methods.

*Gestational age was included in the adjustment.

#### Comparisons between the unicornuate and normal uterine groups

After adjustment, the presence of a unicornuate uterus was associated with an increased risk of preterm delivery (aOR 2.76, 95% CI 1.33–5.73; p = 0.006) in women with a twin pregnancy. A higher risk of LBW was also noticed, but the difference was not significant. After SR of twins to a singleton, the perinatal outcomes were statistically similar between the unicornuate and normal uterine groups.

## Discussion

In this study, we found that patients with unicornuate uteri who are carrying twins are at increased risk of adverse perinatal outcomes after IVF-ET. However, SR of twins to a singleton yielded much more satisfactory perinatal outcomes. These findings suggest that effort should be made to minimize the risk of twin pregnancies in patients with unicornuate uterine anomaly undergoing IVF-ET.

Unicornuate uterus is caused by the non-development of one Müllerian duct, either partially or completely^14^. Most patients with unicornuate uteri remain asymptomatic, and this abnormality is usually incidentally detected in infertility examinations^5^. However, the relationship between unicornuate uterine anomaly and infertility remains controversial^15,16^. In this study, the causes of infertility among the 206 enrolled patients included female factors (n = 121), male factors (n = 8), combined factors (n = 74) and unknown factors (n = 3), suggesting that infertile patients with this uterine anomaly are always complicated with other infertility causes.

The challenge faced by patients with a unicornuate uterine anomaly has long been thought to be pregnancy maintenance rather than impaired fertility. Patients with this uterine anomaly have higher frequencies of spontaneous miscarriage, preterm delivery, abnormal foetal presentation and other similar issues^14^. Diminished muscle mass, abnormal uterine blood flow and cervical incompetence have been proposed as three main aetiologies that explain the poor reproductive performance of the unicornuate uterus^14,17^.

Multiple pregnancies are a common complication of ART, and approximately 98% of multiple pregnancies are twins^8,18^. Additionally, twin pregnancies are associated with significantly higher risks of maternal-infant complications than singleton pregnancies^7,8^. In the present study, the patients with unicornuate uteri who had twin pregnancies had markedly higher risks of adverse outcomes than those with singleton or reduced singleton pregnancies. There was no perinatal mortality in the SR group and only one case of stillbirth, due to umbilical cord factors, in the singleton group (0.7%, (1/139)). However, perinatal mortality increased to 21.7% (20/92) in the twin group, in which there were 5 cases of stillbirth, 4 cases of early neonatal death and 2 cases of 1 foetal death. These outcomes demonstrated the adverse effect of twin pregnancies.

Higher risks of complications have also been reported for twin pregnancies in women with a normal uterus than for singleton pregnancies in such women^19,20^. The comparisons of twin pregnancies between the unicornuate uterine group and normal uterine group in this study showed that the unicornuate uterine anomaly resulted in a poorer tolerance for twin pregnancies and may result in more negative outcomes than a normal uterus, which was consistent with our previous study^11^. In patients with unicornuate uteri, gestational capacity is jeopardized by the presence of only one-half of the full uterine musculature, and the myometrium thickness continues to diminish during pregnancy, which may cause inconsistencies and poor tolerance as gestation advances^14,21^.

In this study, for women with a unicornuate uterus, SR of twins to a singleton resulted in a live birth rate of 100% and significantly reduced the risks of preterm delivery and LBW to levels equivalent with those for reduced singletons in the normal uterine group. These outcomes suggested that SRs carried out by experienced operators are safe and that women with a unicornuate uterus have better reproductive performance when twins are reduced to singletons. However, in our previous study, the singleton pregnancy outcomes were more satisfactory in the normal uterus group than in the unicornuate uterus group. The difference in these results may be attributable to the small size of the unicornuate uterus group (especially the SR group) in this study. Additionally, only perinatal pregnancies were considered here, and the outcomes of SR may differ from those for primary singletons. Regardless, these results suggested that for a unicornuate uterus carrying a twin pregnancy, SR to a singleton could be considered a means to improve perinatal outcomes. However, SR is definitely not an ideal path for multiple pregnancies. The appropriate action is to minimize the risk of multiple pregnancies by addressing the root cause via single-embryo transfer (SET).

SET has been reported to have a similar cumulative clinical pregnancy rate as multiple ETs and significantly reduced risks of multiple pregnancies and maternal-infant complications^22^. However, in China, couples are eager to have two babies in one delivery due to the family planning policy (which was changed in 2016); thus, double or triple ETs are preferred. In the present study, the data were collected from 2012 to 2014; 175 out of the 206 unicornuate uterine patients (85.0%) received double ETs, and 2 patients (1.0%) received triple ETs, which may be the main cause of the multiple pregnancies and subsequent SRs. Although our hospital discontinued triple or more ETs in 2015, routine double ETs should also be undertaken with caution for women with a normal uterus and should be banned for patients with high-risk unicornuate uterine anomaly.

The rarity of the unicornuate uterus caused the relatively small sample size to be one limitation and limited us from obtaining more convincing results. A further prospective study with a larger sample size will be helpful for analysing these outcomes more objectively. Another limitation was that all pregnancy outcomes were obtained via telephone calls or fax, and some details were difficult to collect. In addition, due to ethical concerns about SR, randomized controlled trials are difficult to perform. Moreover, although some units routinely offer chorionic villous sampling (CVS) before SR, considering the concomitant risks of contamination and miscarriage^23^, CVS was not routinely offered in our centre.

## Conclusions

The presence of unicornuate uterine anomaly is associated with increased risks of adverse perinatal outcomes for twin pregnancies. However, the SR of twins to a singleton pregnancy produces similar perinatal outcomes in unicornuate and normal uteri. Therefore, the risk of twin pregnancies should be minimized in patients with unicornuate uterine anomaly undergoing IVF-ET.

## Data Availability

The datasets generated during and/or analysed during the current study are available from the corresponding author on reasonable request.
